# Potentially Novel *Ehrlichia* Species in Horses, Nicaragua

**DOI:** 10.3201/eid2102.140290

**Published:** 2015-02

**Authors:** Victoria L. O’Nion, Hernan J. Montilla, Barbara A. Qurollo, Ricardo G. Maggi, Barbara C. Hegarty, Susan J. Tornquist, Edward B. Breitschwerdt

**Affiliations:** Oregon State University, Corvallis, Oregon, USA (V.L. O’Nion, H.J. Montilla, S.J. Tornquist);; North Carolina State University, Raleigh, North Carolina, USA (B.A. Qurollo, R.G. Maggi, B.C. Hegarty, E.B. Breitschwerdt)

**Keywords:** bacteria, tickborne pathogen, Anaplasmataceae, Ehrlichia, equine, horse, PCR, DNA sequencing, Central America, Nicaragua

## Abstract

*Ehrlichia* sp. DNA was amplified from 4 *Ehrlichia*-seroreactive horses from Mérida, Nicaragua. Sequencing of 16S rDNA, *sodB,* and *groEL* genes indicated that the bacterium is most likely a novel *Ehrlichia* species. The tick vector and the potential for canine and human infection remain unknown.

Worldwide, ehrlichioses are considered emerging infectious diseases of animals and humans. Transmitted by ticks, ehrlichae are obligate intracellular, gram-negative bacteria that infect animals and humans (*1*). Recognized species include *E. canis, E. chaffeensis, E. ewingii, E. muris,* and *E. ruminantium* (*1*). Other species identified in North America are Panola Mountain (*2*) and the *E. muris*–like agent (*3*). Two recently identified new species are *Ehrlichia* sp. AvBat, isolated from *Argas vespertilionis* ticks in France (*4*), and *E. mineirensis*, isolated from hemolymph of *Rhipicephalus microplus* ticks in Brazil (*5*). 

Although studies from Brazil and Oklahoma (USA) have documented reactivity to *Ehrlichia* spp. (*6*,*7*) in horse serum, no reports have documented isolation or PCR detection of *Ehrlichia* spp. infection in horses worldwide. In North America, cervids are reservoir hosts for *E. chaffeensis,* which after tick transmission causes monocytic ehrlichiosis in humans (*1*). Although equids are not known hosts for *E. chaffeensis,* bacterial DNA has been amplified from ticks (*Dermacentor nitens* and *Amblyomma cajennense*) collected from horses in Panama (*8*). *Anaplasma phagocytophilum* and *Borrelia burgdorferi,* causes of granulocytic anaplasmosis and borreliosis (Lyme disease), respectively, are transmitted by *Ixodes scapularis* and *I. pacificus* ticks in North America and infect cats, dogs, horses, and humans.

In Mérida, Nicaragua, the potential for infection of horses by tickborne pathogens is a concern because of the horses’ often poor body condition and heavy tick infestations. In 2013, to determine exposure of equids to >1 tickborne organism, visiting veterinary students collected blood samples from horses. 

## The Study

With approval from the Oregon State University Animal Care and Use Committee (Animal Care and Use Proposal no. 4329), blood samples were collected from 92 horses being evaluated for medical conditions (e.g., anorexia, weight loss, lameness, administration of endoparasiticides and ectoparasiticides) or before elective surgery (e.g., castration, wound repair) at the clinic in Mérida from August 28 through September 4, 2013. After jugular venipuncture, 6 mL of blood was collected into EDTA tubes.

Each whole blood sample was tested for antibodies against *Anaplasma* spp. (*A. phagocytophilum* and *A. platys*), *B. burgdorferi* sensu stricto, and *Ehrlichia* spp. (*E.canis, E. chaffeensis,* and *E. ewingii*) by using the ELISA-based assay SNAP 4DxPlus (IDEXX Laboratories, Inc., Westbrook, ME, USA) according to the manufacturer’s instructions (*9*). The assay does not use a host species–specific conjugate and can therefore be used in research to screen mammals other than dogs. According to assay results, 51 (55%) horse serum samples were *Ehrlichia* spp. seroreactive. One sample was *B. burgdorferi* seroreactive, whereas none were *Anaplasma* spp. seroreactive. 

The 51 *Ehrlichia* spp.–reactive serum samples were subsequently stored at 28°C for up to 1 week during transport to the United States. For PCR testing, samples were shipped (US Department of Agriculture import permit no. 13846) to the Intracellular Pathogens Research Laboratory, Center for Comparative Medicine and Translational Research, North Carolina State University, College of Veterinary Medicine. DNA extraction from 200 μL of EDTA-anticoagulated whole blood was performed by using a QIAsymphony DNA Mini Kit (QIAGEN, Valencia, CA, USA; catalog no. 931236). Previously described PCRs were used to amplify a 420-bp fragment of the *16S* rRNA gene, a 620-bp fragment of the *GroEL* gene, and a 300-bp fragment of the *Ehrlichia* spp. *sodB* gene (*2*,*10*,*11*). A larger, 600-bp, fragment of the *sodB* gene was amplified from 1 sample that was positive by PCR by using the following unpublished primers: sodbEhrl600-F 5′-ATGTTTACTTTACCTGAACTTCCATATC-3′ and sodbEhrl600-R 5′-ATCTTTGAGCTGCAAAATCCCAATT-3′. Positive (*Anaplasma* or *Ehrlichia* spp. plasmid DNA) and negative (RNase-free molecular-grade water and a DNA extraction control consisting of uninfected canine genomic DNA) controls were used for each assay. Amplified DNA was sequenced directly by GENEWIZ, Inc. (Research Triangle Park, NC, USA), and alignments were compared with those of GenBank sequences by using AlignX software (Vector NTI Advance version 11.5; Invitrogen, Carlsbad, CA, USA).

Of the 51 samples tested by *16S* rDNA PCR, the rDNA amplicon sequences were identical for 4 (8%). Sequence comparisons of the amplified products with *Ehrlichia* spp., *Anaplasma* spp., and *Neorickettsia risticii* sequences in GenBank are summarized in Table. Identical *GroEL* and *sodB* DNA sequences were amplified from 3 of 4 horses.

**Table:** Base pair similarities for DNA sequences of novel *Ehrlichia* species obtained from horses in Nicaragua in 2013*

Bacteria	GenBank accession nos.†	Gene, no. positive/no. tested (%)		
		*16S* rRNA	*GroEl*	*SodB*
*E. ruminantium*	CR925678	361/374 (96.5)	534/590 (90.5)	508/599 (84.8)
*E. canis*	CP000107	356/374 (95.2)	540/590 (91.5)	496/599 (82.8)
*E. chaffeensis*	CP000236	360/374 (96.2)	529/590 (89.7)	491/599 (82.0)
*E. ewingii*	NR_044747, AF195273, KC778986	352/374 (94.1)	530/590 (89.8)	241/306 (78.8)
*A. marginale*	CP006847	339/374 (90.6)	417/590 (70.8)	348/599 (58.1)
*A. phagocytophilum*	CP006618	348/374 (93.0)	439/590 (74.4)	367/599 (61.2)
*N. risticii*	CP001431	306/374 (81.8)	395/590 (66.9)	358/599 (59.8)

*Sequences for partial *16S* rRNA, *GroEl*, and *SodB* genes are compared with GenBank database sequences from *Ehrlichia ruminantium, E. canis, E. chaffeensis*, *E. ewingii, Anaplasma marginale, A. phagocytophilum*, and *Neorickettsia risticii.* †GenBank sequence accession numbers for *16S* rRNA, *GroEl,* and *SodB* for *Ehrlichia* sp. from horses in Nicaragua are KJ434178, KJ434179, and KJ434180, respectively.

## Conclusions

According to serologic, PCR amplification, and DNA sequencing results, tick-infested horses in Mérida, Nicaragua, might be infected with a potentially novel *Ehrlichia* species. Initial serologic screening with the rapid ELISA indicated that exposure to >1 *Ehrlichia* species is common among horses in Nicaragua (55%). For dogs, SNAP 4DxPlus results can be positive after exposure to *E. canis, E. chaffeensis, E. ewingii*, and potentially *E. muris* and Panola Mountain ehrlichiae *(**2*,*3*,*6**).* Before this study, equine exposure to *A. phagocytophilum* (previous designation *E. equi*) in Nicaragua was considered more likely because equine exposure has occurred in Guatemala; this rickettsial organism is pathogenic for cats, dogs, horses, and humans (*12*). However, in North America, *A. phagocytophilum* is transmitted by *I. scapularis* and *I. pacificus* ticks*,* which have not been reported in Nicaragua. In future studies of horses in Nicaragua, ticks will be collected for identification. In Guatemala, *R. microplus* and *A. cajennense* ticks were the predominant species found on cattle, whereas *D. nitens* and *A. cajennense* ticks were most commonly found on horses (*12*). Of note, in Guatemala, tick infestation levels were substantially higher and body condition scores lower for horses than for cattle. Also, cattle were exposed to an agent with serologic cross-reactivity and close genetic relatedness to *E. ruminantium.*

The partial *16S* rDNA sequences obtained from these horses most likely represent a novel species of *Ehrlichia*. This conclusion is further supported by sequence analysis of 2 protein-coding genes, *sodB* and *groEL*. Partial sequences from *sodB* and *groEL* genes demonstrated similarity to *Ehrlichia* spp. sequences found in GenBank, but they were not 100% identical to any sequences deposited to date. When *16S* rDNA for rickettsiae are compared, Fournier et al. recommend that gene homology for organisms of identical species and genus be 99.8% and 98.1%, respectively (*13*). Although identical species are typically defined as being >99% identical with a reference sequence, the percentage identity needed to define a separate species is debated, ranging from 97% to 99.5% (*14*). Calculation of values can be based on alignment methods, reference databases, and number of basepairs in the sequence. Fournier et al. recommend that for novel species identification and rickettsiae classification, protein-coding genes should be used, specifically the 4 protein-coding genes *gltA*, *ompA*, *ompB,* and gene D in addition to the *16S* rRNA gene. To date, *16S* rDNA sequences or whole-genome sequencing have been used to classify *Ehrlichia* species and strains. Collectively, and as depicted in the Figure (in which phylogenetic alignment trees for all 3 genes tested in this study were constructed by using reference sequences from representative members of the genera *Anaplasma*, *Ehrlichia*, and *Neorickettsia*), the genetic findings in this study support infection of horses in Nicaragua with a novel *Ehrlichia* species. To confirm this possibility, future efforts will focus on cell culture isolation of the *Ehrlichia* organism from horses in Nicaragua.

**Figure:**
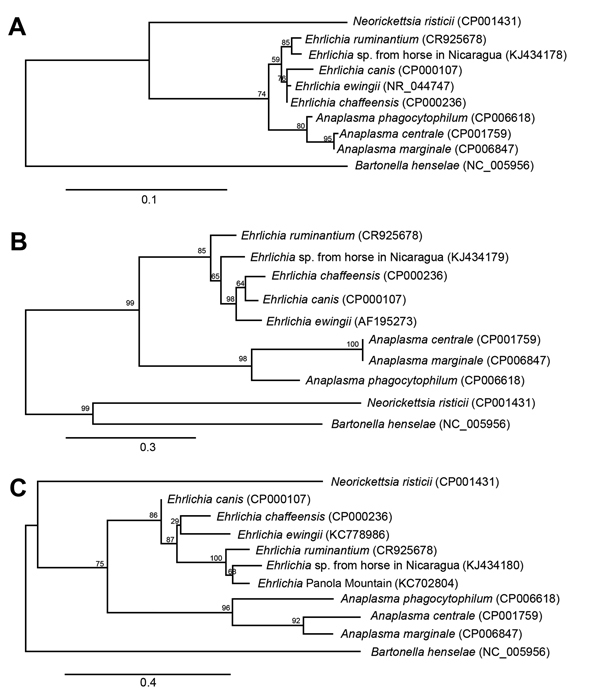
Phylogenetic trees of *Ehrlichia* sp. from horses in Nicaragua and selected bacterial species (GenBank accession numbers for reference sequences in parenthesis) based on partial sequences from genes coding for 16SrRNA (A), GroEL (B), and SodB (C). Sequences were aligned by using MUSCLE version 3.7 (http://www.ebi.ac.uk/Tools/msa/muscle/), and alignments were refined by using Gblocks version 0.91b (http://www.idtdna.com/gblocks.com). Phylogenetic trees were constructed by using PhyML version 3.0 aLRT (http://code.google.com/p/phyml/) under the HKY85 model, and the resulting trees were rendered by using TreeDyn version 198.3 (http://www.treedyn.org/). Scale bars indicate number of substitutions per site, and the numbers in the branches represent percentage support of the node.

Vectorborne pathogens can infect any host species bitten by infected ticks. At least 4 *Ehrlichia* species have been implicated as being pathogenic for canids and humans (*1*–*3*). Thus, future studies should also determine whether dogs, other animals, and humans in Nicaragua are exposed to and infected with this potentially novel *Ehrlichia* species.
